# Genistein ameliorates non-alcoholic fatty liver disease in mice by modulating gut microbiota and hepatic macrophage polarization

**DOI:** 10.1038/s41598-025-27783-3

**Published:** 2025-12-18

**Authors:** Shanshan Fang, Hui Han, Rui Wang, Siyu Li, Rui Yang, Shomaila Mehmood, Qiang Jia

**Affiliations:** 1 School of Basic Medicine, Bengbu Medical University, Bengbu, 233030 China; 2 School of Biology and Food Engineering, Hefei Normal University, Hefei, 230601 China; 3 Huaibei People’s Hospital, Huaibei, 235000 China; 4 Institute of Environmental Health Sciences, Wayne State University, Detroit, 48201 USA

**Keywords:** Genistein, Non-alcoholic fatty liver disease, Chronic inflammation, Macrophage polarization, Gut microbiota, Diseases, Gastroenterology, Microbiology

## Abstract

Genistein (GEN) exhibits diverse biological activities. This study is conducted to explore the effect of GEN on metabolic dysfunction-associated steatotic liver disease (MASLD) mice. MASLD was induced in mice by a high-fat diet (HFD). The results indicated that GEN significantly reduced HFD-induced body weight and improved lipid homeostasis and glucose tolerance in HFD-fed mice. Furthermore, GEN intervention markedly ameliorated hepatic pathological changes, hepatic dysfunction, and macrophage polarization. Meanwhile, GEN had a beneficial significant impact on colonic pathological changes and the expression of tight junction-related proteins. The results of gut microbiota showed that high-dose GEN intervention was able to down-regulate Firmicutes and Proteobacteria and increase Bacteroidota at the phylum level. At the genus level, GEN intervention increased *Lactobacillus*, *Muribaculum*, and *Muribaculaceae_unclassified* in MASLD mice, and decreased *Ruminococcus*, *Allobaculum*, and *Helicobacter*. In conclusion, GEN attenuated lipid metabolism disorders and hepatic inflammatory reaction *via* regulating the composition of the gut microbiota and hepatic macrophage polarization in MASLD mice.

**Supplementary Information:**

The online version contains supplementary material available at 10.1038/s41598-025-27783-3.

## Introduction

Non-alcoholic fatty liver disease (NAFLD) is mainly caused by obesity and metabolic dysfunction that has emerged as a prevalent global liver disease^1^. With the progressive advancement in the understanding of the pathogenesis of NAFLD, the terminology has been updated to metabolic dysfunction-associated steatotic liver disease (MASLD), while non-alcoholic steatohepatitis (NASH) has been redefined as metabolic dysfunction-associated steatohepatitis (MASH). The early stage of MASLD affects more than 5% of the liver parenchyma, although in the absence of obvious hepatocellular injury. If fat accumulation is not adequately controlled, it will trigger inflammatory responses, and accelerate the progression of hepatocyte injury^2^. This process accelerates the progression of MASLD, beginning with steatosis and advancing to MASH, which evolves into irreversible outcomes including liver fibrosis, cirrhosis and liver cancer^3^. So far, no widely accepted and effective treatments have been reported for MASLD^4^.

As central players in metabolic syndrome, liver macrophages exert crucial effects through their polarization, particularly in improving glucose tolerance, alleviating insulin resistance (IR), and reducing hepatic lipid accumulation and damage^5,6^. Inducible nitric oxide synthase (iNOS) and arginase-1 (Arg1) are generally employed for the differentiation between M1 and M2 macrophage phenotypes^7^. In the advanced stages of MASLD, hepatic fat accumulation recruits macrophages. M1 macrophages are activated by microbial lipopolysaccharides (LPS), cytokines, and other factors; upon activation, M1 macrophages release a substantial amount of cytokines, including tumor necrosis factor-alpha (TNF-α), which further induces chronic liver inflammation and exacerbates the progression of MASLD^8^. M2 macrophages are activated by immunoregulatory signals and produce immunoregulatory factors such as Arg1 and interleukin-10 (IL-10). M2 macrophages function to attenuate inflammatory response and thereby promote liver tissue repair. The polarization of M1/M2 macrophages is indeed dynamic, and therefore, activating M2 macrophages to replace M1 macrophages represents a critical pathway for reducing hepatic lipid accumulation and inflammation, thus ultimately ameliorating MASLD.

The gut and liver have a close anatomical and functional relationship *via* the portal vein system, which is collectively denoted as the gut-liver axis. Around 70% of liver blood supply originates from the intestine, which facilitates the transport of dietary components and gut microbiota and its metabolites from the gut to the liver through the portal vein system^9^. Research conducted in both human subjects and animal models has highlighted the gut microbiota and their metabolites to influence further progression of MASLD *via* a diverse array of mechanisms^10,11^. A high-fat diet (HFD) often causes gut microbiota dysbiosis, thereby disrupting both intestinal barrier morphology and function in mice^12^. LPS, which is present in the outer cell wall of Gram-negative bacteria, is an important exogenous stimulant, also known as endotoxin. LPS can be recognized by pattern recognition receptors, thereby promoting M1 macrophage polarization and exacerbating inflammatory responses. During the development of MASLD, intestinal-derived LPS can permeate the bloodstream through compromised intestinal mucosa, subsequently reaching the liver where they promote macrophage polarization, thereby exacerbating the inflammatory response in the liver^13^. Study has shown that serum LPS is downregulated accompanied by a reduced degree of hepatic inflammation^14^. Accumulating evidence suggests that gut microbiota and injured intestinal mucosa play key roles throughout the progression of MASLD.

Genistein (GEN), as a naturally occurring active isoflavone, is abundantly found in soy products. Accumulating evidence emphasizes its low toxicity and a range of biological activities, such as anti-inflammatory, lipid-lowering, antioxidant, anti-cancer effects^15,16^. GEN modulates gut microbiota composition to ameliorate inflammation responses and IR, thereby inhibiting the release of inflammatory factors. This improvement extends to liver and colon dysfunction in type 2 diabetes mellitus (T2DM) model^17^. GEN also exerts beneficial effects on ulcerative colitis, potentially involving the promotion of M1 to M2 macrophage polarization^7^. Recent studies have shown that GEN can ameliorate the pathological manifestations associated with obesity and MASLD^18–20^. In the current study, we explored the positive effects of GEN on the gut-liver axis, extending mechanistic insights into MASLD mice. Specifically, the present study aimed to explore the potential role of GEN in ameliorating MASLD, and this effect may be mediated by regulating gut microbiota and hepatic macrophage polarization.

## Materials and methods

### Ethics declarations

All animal experiments were conducted following guidelines approved by the Ethics Committee of Bengbu Medical University. Experiments were performed under the approved animal protocol ID 2024 − 198. We adhere to the animal research: reporting of in vivo experiments (ARRIVE) guidelines.

### Animals

All the 48 male C57BL/6J mice, averaging 18 to 20 g in body mass, were sourced from the Animal Center of Anhui Medical University and were housed in an animal room, maintained under standardized laboratory conditions at 22 ± 2 °C, with 12-hour light/dark cycle and a relative humidity ranging from 45 to 55% and the animals were allowed to access food and water.

### Experimental design

After the acclimatization period of 7 days, the mice were randomly divided into 6 groups, with 8 mice per group (4 mice in each cage): normal diet (standard laboratory rodent diet) group (NOR), HFD (60% of energy from fat, Xietong Pharmaceutical Bio-engineering Co., Ltd, Nanjing, China) group, HFD + 30 mg/kg GEN (Macklin Biochemical Technology Co., Ltd, Shanghai, China) group (LGE), HFD + 60 mg/kg GEN group (MGE), HFD + 120 mg/kg GEN group (HGE), HFD + 100 mg/kg simvastatin group (SIM). The mice in groups LGE, MGE, and HGE were daily gavaged with 30 mg/kg, 60 mg/kg, and 120 mg/kg GEN dissolved in 0.5% carboxymethylcellulose sodium (CMC-Na) for a period of 16 weeks. At the same time, mice in the NOR group and HFD group were daily gavaged with equal amounts of 0.5% CMC-Na solution, while mice in the SIM group were daily gavaged with simvastatin dissolved in grade I purified water. The body weight was monitored on a weekly basis.

### Oral glucose tolerance test

On the penultimate day of the experimental period, OGTT was conducted following a 12-hour fasting period in mice. Mice were administered 3 g/kg of glucose solution *via* gavage^21^. Subsequently, the levels of blood glucose were assessed at various intervals (0, 30, 60, 90, and 120 min) from tip of the tail vein in mice. The measurements were performed using a Sinocare Safe-Accu glucometer (Sinocare, Hunan, China) and the areas under the curve (AUC) were calculated.

### Liver ultrasound imaging observation

After the OGTT assay, the mice were anesthetized with isoflurane and subjected to ultrasound analyses to capture B-mode images of the liver and kidneys. After obtaining the images, markings were made to delineate the liver and kidney areas, avoiding bile ducts and mesenteric tissue. Circular regions of interests (ROIs) were selected in the liver images and in the renal cortex region of the kidney images. Echo values of the liver and kidney were analyzed separately. Subsequently, the sonographic hepato-renal index (SHRI) was calculated as SHRI = liver echo value / kidney echo value for further statistical analysis^22^.

### Biochemical analysis

The mice were anesthetized with isoflurane. Subsequently, murine blood was obtained through the retro-orbital sinus followed by cervical dislocation. The collected blood was centrifuged at 3000 revolutions per minute (rpm) for 15 min using a centrifuge. After that, the serum was collected, aliquoted, and stored in a -80℃ freezer for the determination of various biochemical indicators. The mice were subjected to thorough dissection during which the liver, colon, and epididymal white adipose tissue (eWAT) were promptly excised, accurately weighed, and subsequently subjected to further analysis.

Following the manufacturer’s instructions, the assessment of serum and liver biochemical indices were evaluated in order to determine the status of lipid metabolism and hepatic functionality. The above tests were performed using standardized assay kits (Nanjing Jiancheng Institute of Bioengineering, Nanjing, China).

Serum LPS was measured using a commercial kit (Ruixin Biotechnology, Quanzhou, China). Using the standard concentration as the ordinate and the OD value as the abscissa, the standard curve equation was obtained through four - parameter Logistic curve fitting. Based on the OD values of serum samples, this equation was used to calculate the concentration of LPS in the tested samples.

### Morphological examination

The livers, colons, and eWAT of mice were processed as follows: the tissues were washed with a pre-chilled saline solution, dried with filter paper to remove excess moisture, and then weighed. The liver and colon were immersed in 4% paraformaldehyde (PFA), while the eWAT was fixed in a fat-fixing solution. Following fixation, the specimens were dehydrated and subsequently embedded in wax, then sectioned and stained using Hematoxylin and Eosin (H&E) staining for general tissue morphology and Oil Red O for lipid accumulation.

All regions of each liver slice were examined by an experienced pathologist. The NAFLD activity score (NAS) was graded based on hepatic steatosis, lobular inflammation, and hepatocyte ballooning. Each liver section was scored according to previously published research^18^.

### Western blot analysis

20 mg of liver tissue was homogenized with an appropriate amount of lysis buffer mixed with PMSF. Following centrifugation, the resultant supernatant was recovered and preserved. The BCA method was utilized to quantify the protein concentration. In each sample mixture, containing equal amounts of proteins, separation was carried out using 10% SDS-PAGE gels, followed by transferring the isolated proteins to PVDF membranes. The PVDF membranes containing the isolated proteins were blocked using 3% bovine serum albumin (BSA), washed using TBST, and incubated overnight with corresponding primary antibodies: iNOS (1:4000, Proteintech, Wuhan, China), TNF-α (1:500, Affinity Biosciences, Changzhou, China), IL-10 (1:500, Affinity), Arg1 (1:50000, Proteintech), and β-actin (1:3000, Servicebio, Wuhan, China) at 4 °C. The next day, the washed membranes were incubated with anti-rabbit antibody (1:3000, Servicebio). Images were captured with enhanced chemiluminescence and the band intensities were quantified after normalizing them to β-actin using Image J (1.46r) software.

### Immunohistochemistry (IHC)

Firstly, paraffin-embedded colon tissue sections were treated with xylene to remove the paraffin. The tissues were rehydrated through a graded ethanol series with descending concentrations. Subsequently, hydrogen peroxide treatment was applied to neutralize peroxidase activity. The colon tissue sections were incubated with primary antibodies against claudin-1 (1:100, Servicebio), occludin (1:100, Servicebio) following blocking with BSA. Subsequently, secondary anti-rabbit antibodies (1:200, Servicebio) were applied, followed by staining with diaminobenzidine and hematoxylin. The results were quantified according to the previous study^7^.

### Analysis of intestinal bacterial community

Fecal specimens were randomly gathered from 5 mice in each experimental group and microbial DNA was isolated from each murine fecal sample utilizing the E.Z.N.A.^®^ Stool DNA Kit (Omega Bio-Tek, Norcross, USA). The quality of the DNA was evaluated using agarose gel electrophoresis while its concentration was determined using a NanoDrop spectrophotometer (Thermo Scientific, New York, USA). Specific primers were used to amplify distinct regions (V3-V4) of the bacterial 16 S rRNA genes following the protocol by Chen et al.^21^. Samples were sequenced and analyzed according to the manufacturer’s instructions (LC-Bio Technology Co., Ltd., Hangzhou, China).

### Statistical analysis

Statistical analysis was conducted using GraphPad Prism version 9.5.1, and the data were presented as mean ± standard deviation (SD). Two-way analysis of variance (ANOVA) was used to assess body weight differences, while one-way ANOVA was used to assess others statistical differences among multiple groups.

## Results

### Effects of GEN on body weight, OGTT, and lipid profiles in HFD-fed mice

**Fig. 1:**
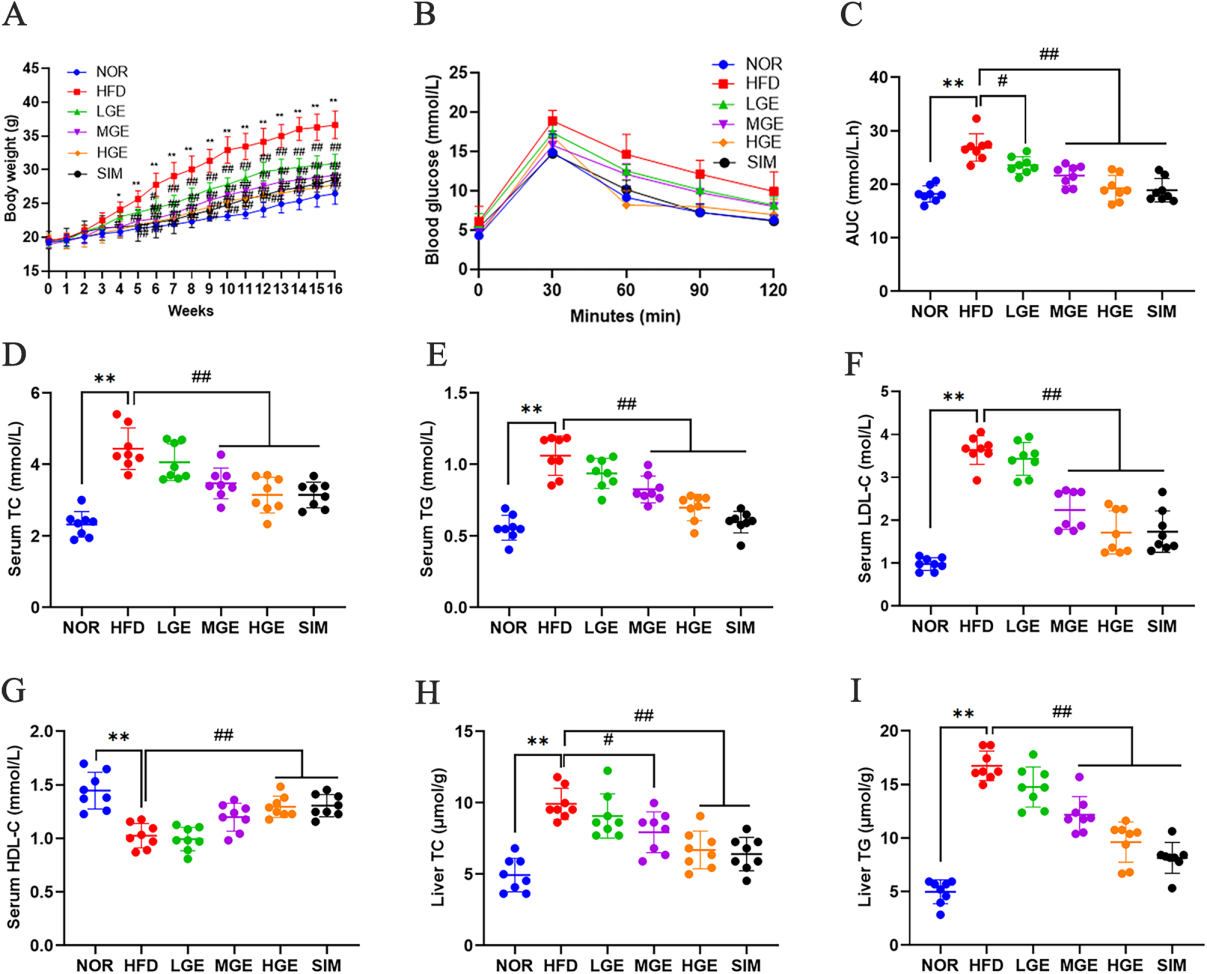
GEN ameliorated body weight gain, glucose tolerance, and lipids profiles. (**A**) Body weight; (**B**) OGTT; (**C**) AUC; (**D**) Serum TC level; (**E**) Serum TG level; (**F**) Serum LDL-C level; (**G**) Serum HDL-C level; (**H**) Liver TC level; (**I**) Liver TG level. Values are mean ± SD (*n* = 8). *P < 0.05, **P < 0.01 versus NOR group; #*P* < 0.05, ##*P* < 0.01 versus HFD group.

As depicted in Fig. 1A, during the experiment, despite the absence of significant alterations among groups in the first three weeks, there was a marked upward curve observed in the body weight of the HFD mice during the fourth week, compared to the NOR mice. The mice treated with GEN showed a significant decline in body mass, in a time- and dose-dependent manner. The HGE group exhibited a body weight gain similar to that of the SIM group. These results indicated that GEN inhibited body weight gain in HFD-fed mice.

As depicted in Fig. 1B-C, the OGTT data indicated that the blood glucose levels of all mice reached their peak within 30 min after gastric glucose administration. Compared with the NOR mice, HFD-fed mice exhibited consistently higher blood glucose levels at all time points, with a slow decline, indicating significantly impaired glucose tolerance. Following GEN intervention, the glucose clearance rates of mice in the GEN treatment groups were significantly improved, accompanied by a marked reduction in AUC (*P* < 0.05 or 0.01), especially in the HGE group. The results suggested that GEN effectively improved glucose tolerance in HFD-fed mice.

The serum lipid profile, as well as the lipid profile within the hepatic tissue showed that compared to the NOR group, the HFD-fed mice exhibited elevated blood fat levels (*P* < 0.01, Fig. 1D-I), with higher serum total cholesterol (TC), triglycerides (TG), low-density lipoprotein cholesterol (LDL-C), and concurrently lower high-density lipoprotein cholesterol (HDL-C) in serum. Both liver TG and TC were also increased. These results indicated prominent hyperlipidemia characteristics in the HFD group. Conversely, the mice treated with MGE and HGE exhibited varying degrees of reduction in abnormally elevated lipid levels (*P* < 0.05 or 0.01) compared to HFD-fed group, although there was no significant difference in the LGE group. Particularly, the HGE group exhibited a lipid-lowering effect similar to that of the SIM group. In addition, these findings indicated that GEN significantly improved lipid metabolism.

### GEN ameliorated liver dysfunction

**Fig. 2:**
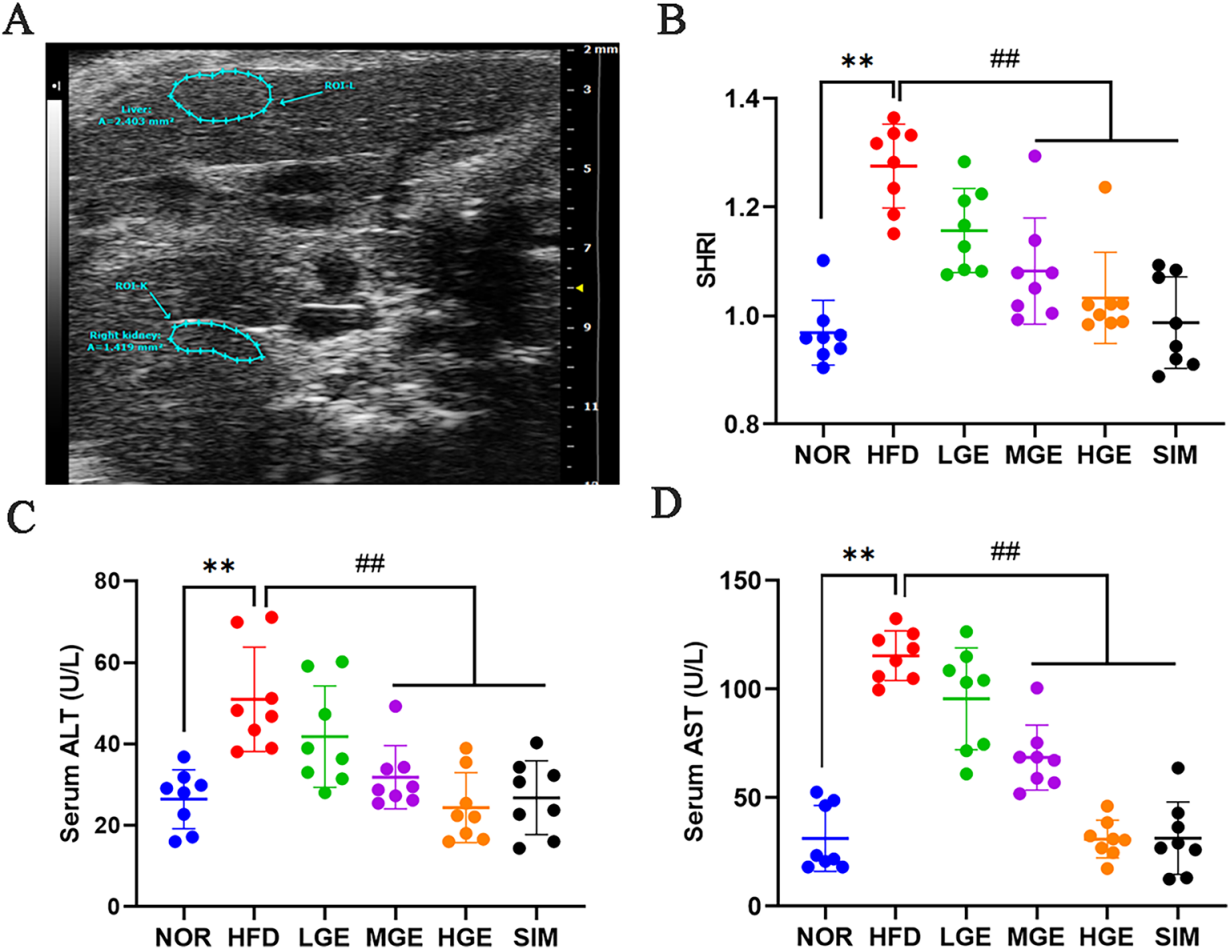
GEN ameliorated liver function. (**A**) The B-mode liver and kidney photograph. To measure the average gray-level intensities for the hepatic and renal parenchyma, the areas of interest ROI-L and ROI-K (blue contour) were meticulously selected; (**B**) SHRI; (**C**) Serum ALT; (**D**) Serum AST. Values are mean ± SD (*n* = 8). **P < 0.01 versus NOR group; ##*P* < 0.01 versus HFD group.

The abdominal ultrasound results of mice indicated that HFD-fed mice had a significantly increased SHRI (*P* < 0.01, Fig. 2A-B) compared with the NOR group, suggesting a marked increase in hepatic fat deposition and aggravation of liver steatosis in HFD-fed mice. Following GEN treatment, mice in the MGE and HGE groups exhibited a significant decline in SHRI (*P* < 0.01, Fig. 2A-B), showing lipid-lowering effects similar to those observed in the SIM group. This suggested that GEN improved the degree of lipid accumulation and steatosis in the liver of MASLD model mice.

Furthermore, compared with the NOR mice, significant increases in serum transaminases (AST, ALT) were observed in HFD-fed mice, indicating impaired liver function in the HFD group. After GEN intervention, abnormal liver function was alleviated to varying degrees (*P* < 0.01, Fig. 2C-D), showing a clear dose-dependent effect of GEN. Particularly, AST and ALT are used as markers of hepatic injury. The levels of these enzymes in the GEN treatment groups were lower than those in the HFD-fed mice, indicating that GEN could ameliorate liver dysfunction in MASLD mice.

### GEN ameliorated hepatic histological changes

**Fig. 3:**
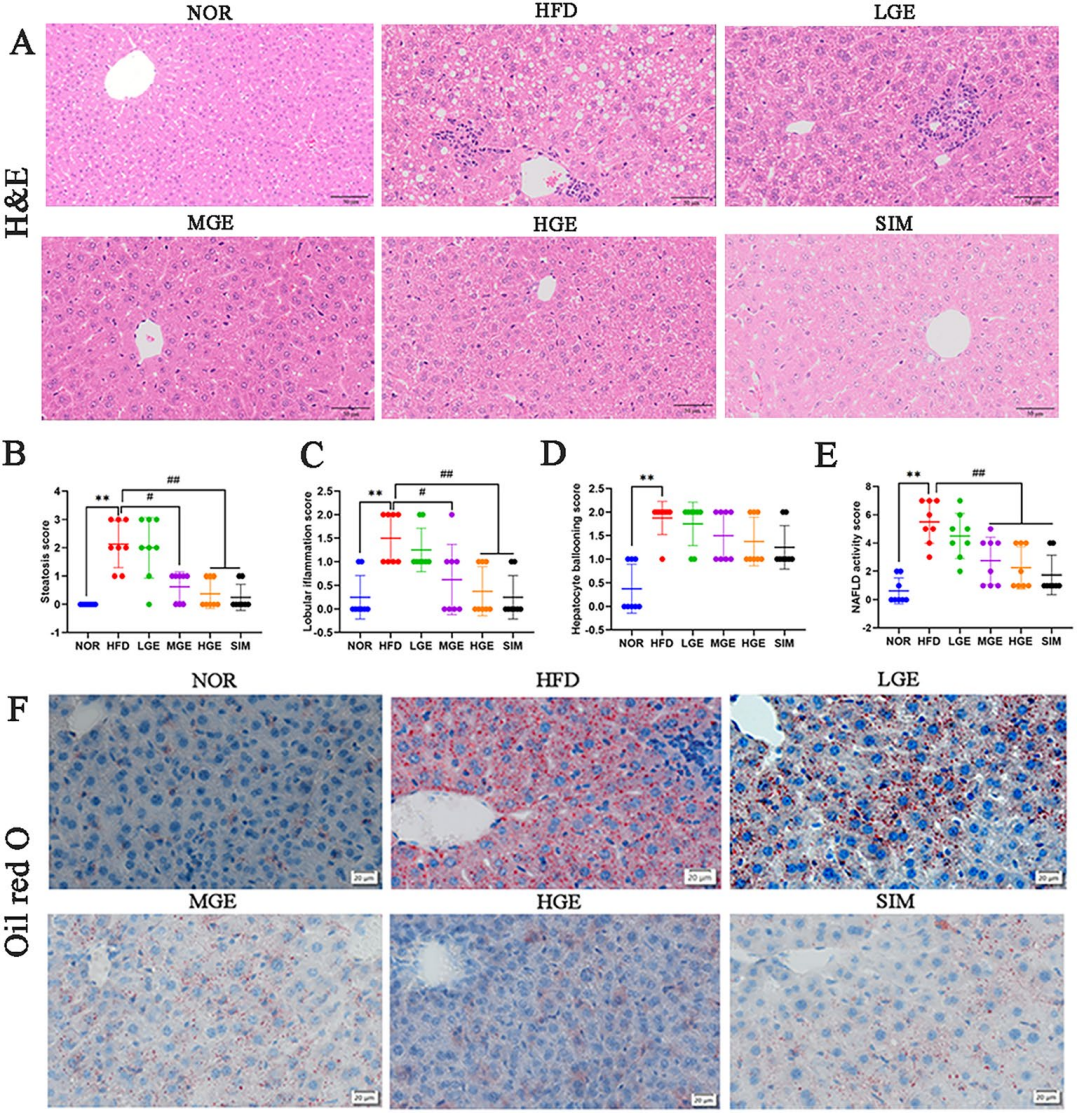
GEN ameliorated liver histopathological alterations. (**A**) H&E. Scale bar = 50 μm. (**B**) Steatosis score; (**C**) Lobular inflammation score; (**D**) Hepatocyte ballooning score; (**E**) NAFLD activity score. Values are mean ± SD (*n* = 8). **P < 0.01 versus NOR group; #*P* < 0.05 versus HFD group; ##*P* < 0.01 versus HFD group. (**F**) Oil red O. Scale bar = 20 μm.

H&E staining (Fig. 3A) demonstrated that HFD-fed mice exhibited hepatocellular ballooning degeneration, accompanied by prominent lipid droplets, unclear cell boundaries, and extensive inflammatory cell infiltration, indicating severe features of MASLD. By contrast, liver damage in the SIM group showed significant improvement, with a noticeable decrease in lipid vacuoles. GEN intervention protected against hepatic steatosis and significantly suppressed inflammation. Particularly, the HGE group showed improvements similar to those of the SIM group, manifested as effective improvement of hepatocellular ballooning degeneration and normalization of hepatic cords and sinusoidal structures. As depicted in Table 1; Fig. 3B-E, the HFD group had higher histological scores for steatosis, inflammation and hepatocyte ballooning than the NOR group, and GEN administration significantly reduced the NAS relative to the HFD group. These findings demonstrated that GEN could ameliorate hepatic pathological damage in mice fed with HFD.

**Table 1:** NAFLD histopathological scores.

Group	*N*	Steatosis					Lobular inflammation					Hepatocyte ballooning		
		0	1	2	3		0	1	2	3		0	1	2
NOR	8	8	-	-	-		6	2	-	-		5	3	-
HFD	8	-	2	3	3		-	4	4	-		-	1	7
LGE	8	1	3	3	1		-	6	2	-		-	2	6
MGE	8	3	5	-	-		4	3	1	-		-	4	4
HGE	8	5	3	-	-		5	3	-	-		-	5	3
SIM	8	6	2	-	-		6	2	-	-		-	6	2

Data showed the distribution of mice with specific histopathological grades in each experimental group. By Brunt’s classification, hepatic steatosis was graded as 0 (< 5%), 1 (< 33%), 2 (33–66%), and 3 (> 66%); the inflammatory response was divided into four grades: 0 (normal), 1 (mild), 2 (moderate), and 3 (severe); hepatocyte ballooning was quantified by a three - tiered grading system: 0 (no ballooning), 1 (few balloon cells), and 2 (abundant prominently ballooned cells).

Oil Red O staining of hepatic sections was shown in Fig. 3F, where the red areas represented lipid droplets in the liver. Compared with the NOR mice, the red-stained areas in HFD-fed mice occupied most of the field of view, indicating a marked increase in lipid aggregation. However, significant improvements were observed in the GEN intervention groups and SIM group, further indicating that GEN could reduce HFD-induced hepatic fat deposition.

### GEN ameliorated the lipid accumulation in eWAT

**Fig. 4:**
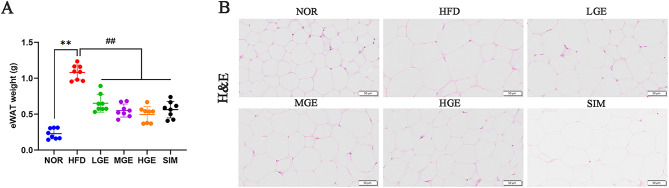
GEN ameliorated eWAT weight gain and histomorphologic changes. (**A**) eWAT weight. Values are mean ± SD (*n* = 8). **P < 0.01 versus NOR group; ##*P* < 0.01 versus HFD group. (**B**) The morphology of adipose tissue. Scale bar = 50 μm.

Compared with the NOR mice, the weight of eWAT was significantly increased (*P* < 0.01, Fig. 4A) in HFD-fed mice. Mice in the GEN treatment groups and SIM group showed a significant decline in eWAT weight. These results indicated GEN could inhibit weight gain of eWAT induced by HFD in mice.

H&E staining was performed on histological sections of eWAT. As shown in Fig. 4B, compared with normal mice, the adipocytes in HFD-fed mice were notably enlarged and distorted, indicating excessive fat accumulation induced by HFD. Following intervention with GEN and simvastatin, the enlargement of adipocytes was markedly alleviated, demonstrating that GEN could reduce lipid deposition in eWAT. These findings underscored the potential ameliorative effects of GEN in suppressing eWAT weight gain and mitigating fat accumulation in MASLD models.

### GEN modulated hepatic macrophage polarization

**Fig. 5:**
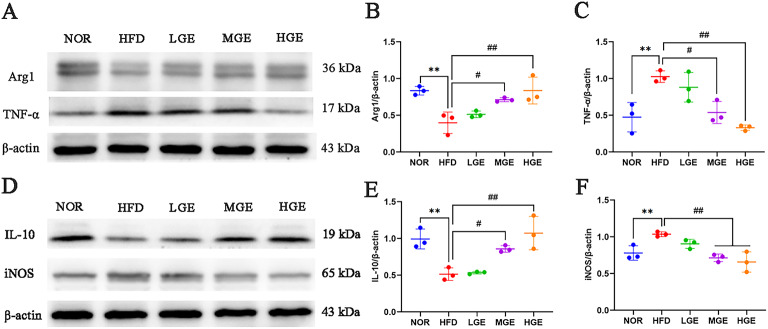
GEN regulated macrophage polarization balance in liver tissue. (**A**) Western blot results of Arg1 and TNF-α; (**B**) Quantification of Arg1; (**C**) Quantification of TNF-α; (**D**) Western blot results of IL-10 and iNOS; (**E**) Quantification of IL-10; (**F**) Quantification of iNOS; Full-length blots are included in the Supplementary Information document. Values are mean ± SD (*n* = 3). **P < 0.01 versus NOR group; #*P* < 0.05, ##*P* < 0.01 versus HFD group.

Western blot analysis was used to detect the polarization levels of hepatic macrophages. As depicted in Fig. 5, compared with the NOR mice, the livers of HFD-fed mice showed a significant increase in the expression of M1 macrophage markers (iNOS and TNF-α) (*P* < 0.01), and a significant decrease in the expression of M2 macrophage markers (Arg1 and IL-10) (*P* < 0.01). By contrast, compared with the HFD group, the HGE and MGE groups exhibited a significant decrease in the expression of M1 macrophage markers (iNOS and TNF-α) (*P* < 0.05 or 0.01), and a significant increase in the expression of M2 macrophage markers (Arg1 and IL-10) (*P* < 0.05 or 0.01). These data indicated that GEN intervention restored the polarization balance of hepatic M1/M2 macrophages in MASLD mice.

### GEN ameliorated colonic barrier function

**Fig. 6:**
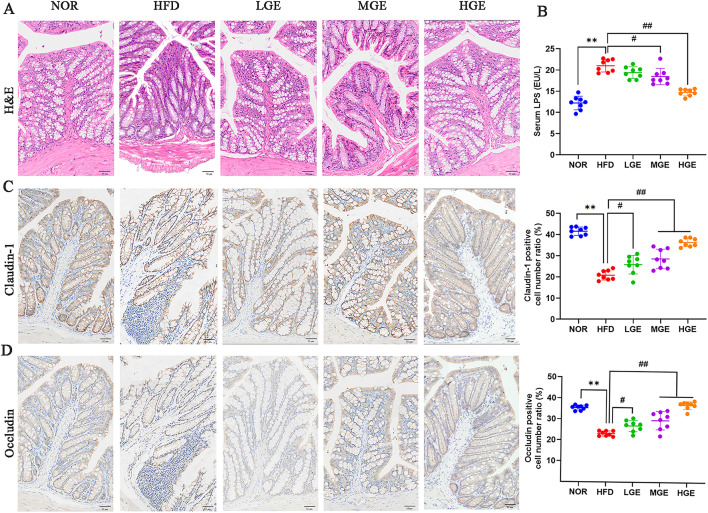
Effect of GEN on colonic histomorphologic changes, serum LPS and colonic mucosa barrier junction. (**A**) H&E; (**B**) LPS; (**C**) IHC staining for Claudin-1; (**D**) IHC staining for Occludin. Values are mean ± SD (*n* = 8). **P < 0.01 versus NOR group; #*P* < 0.05, ##*P* < 0.01 versus HFD group. Scale bar = 50 μm.

As shown in Fig. 6A, the normal colon tissues exhibited abundant crypts and goblet cells. In the HFD group, the colonic tissues showed partial absence of crypts, decreased goblet cells, and inflammatory cell infiltration. The GEN intervention groups showed significant improvement in colon morphology and reduced inflammatory cell infiltration compared with HFD-fed mice. These findings indicated that GEN could inhibit HFD-induced intestinal injury in MASLD mice.

Serum LPS, which often originates from gut microbiota crossing the intestinal barrier, was significantly elevated in HFD-fed mice compared with NOR mice (*P* < 0.01, Fig. 6B), as detected by ELISA. Following GEN intervention, mice in the GEN treatment groups showed decreased serum LPS levels (*P* < 0.05 or 0.01), especially the HGE group. These data suggested that GEN has a down-regulatory effect on serum LPS levels in MASLD mice.

The protein expression of tight junctions in the colon was detected by IHC. Compared with NOR mice, HFD-fed mice exhibited significantly lower expression levels of claudin-1 and occludin (*P* < 0.01, Fig. 6C-D). Conversely, the expression levels of both proteins were elevated in the GEN treatment groups (*P* < 0.05 or 0.01, Fig. 6C-D) compared with those in HFD-fed mice. The result showed that GEN might strengthen colonic epithelial tight junctions and maintain intestinal barrier function in HFD-induced MASLD mice.

### GEN modulated intestinal bacterial community

**Fig. 7:**
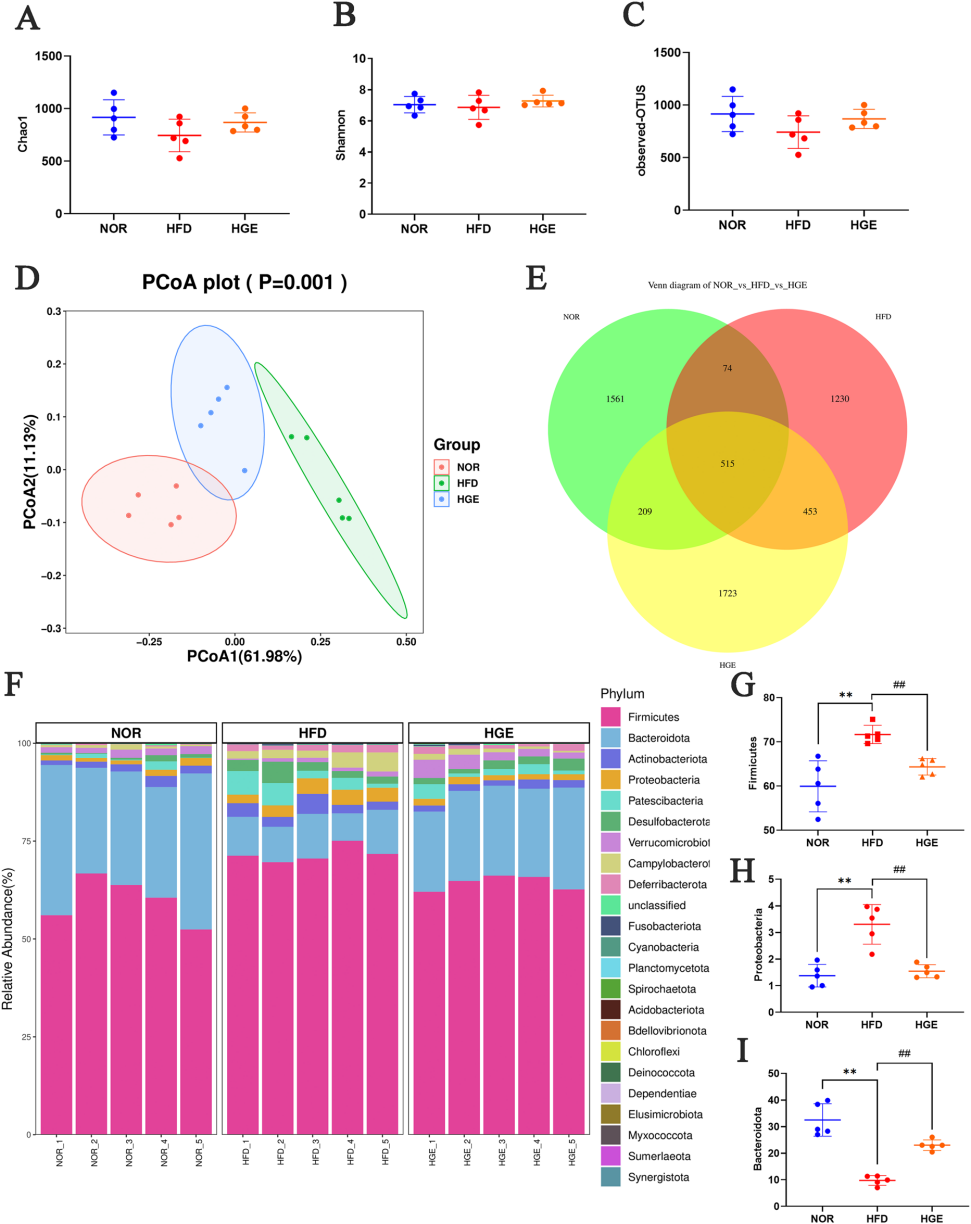
Effect of GEN on the alpha and beta diversity and the relative abundance of gut microbiota. (**A**) Chao1; (**B**) Shannon; (**C**) observed-OTUS; (**D**) PCoA; (**E**) the Venn diagram of NOR-vs-HFD-vs-HGE; (**F**) The abundance of gut micro-biota at the phylum level; (**G**) Firmicutes; (**H**) Proteobacteria; (**I**) Bacteroidota. Values are mean ± SD (*n* = 5), **P < 0.01 versus NOR group; ##*P* < 0.01 versus HFD group.

To determine the protective efficacy of GEN against MASLD, 16 S rRNA high-throughput sequencing was utilized to analyze the gut microbiota composition in murine fecal samples. Firstly, alpha diversity indices (reflecting species richness and diversity) were analyzed. No significant differences were observed in the Chao1, Shannon, and observed operational taxonomic units (OTUs) indices (Fig. 7A-C). These findings indicated there was no significant change in the richness and diversity of gut microbiota among the three groups (NOR, HFD, and HGE groups). Thereafter, principal coordinate analysis (PCoA) was performed to assess beta diversity, which reflected differences in species composition among the three groups. As depicted in Fig. 7D, the NOR group was significantly separated from the HFD group, while the HGE group was located between the NOR and HFD groups and tended towards the NOR group, indicating that GEN intervention could regulate the gut microbiota composition in MASLD mice. Additionally, Venn diagram analysis (Fig. 7E) illustrated the unique and shared OTUs among the three groups. A total of 515 OTUs were identified in all samples, with each group exhibiting a distinct number of detected OTUs. Specifically, the NOR group had 331 more OTUs than the HFD group, and the HGE group had 493 more OTUs than the HFD group. These data indicated that GEN could mitigate the HFD-induced decrease in OTU count, thereby exerting a regulatory effect on the gut microbiota in MASLD mice.

**Fig. 8:**
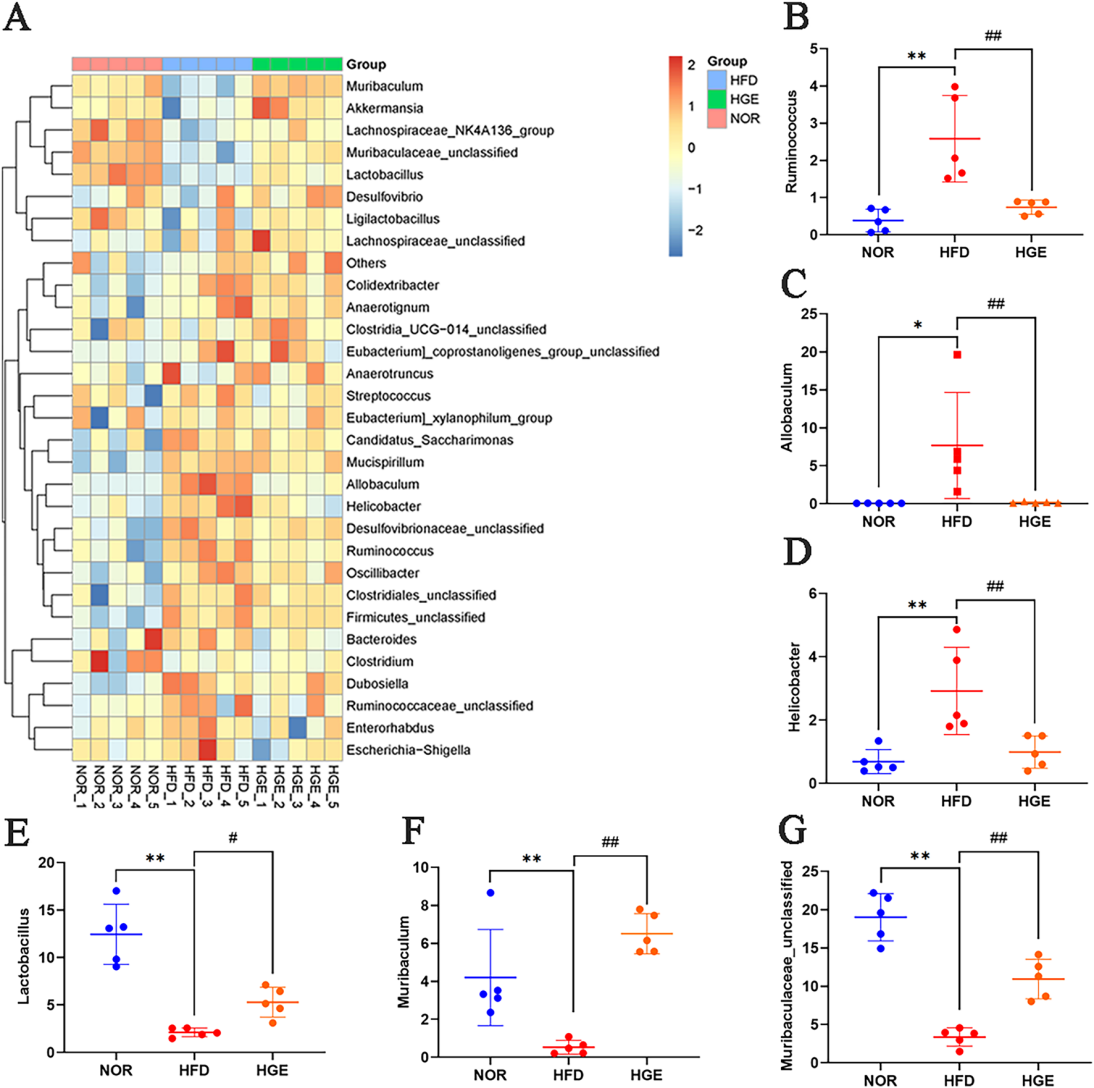
Effect of GEN on the abundance of gut microbiota at the genus level. (**A**) The genus heatmap of gut microbiota; (**B**) *Ruminococcus*; (**C**) *Allobaculum*; (**D**) *Helicobacter;* (**E**) *Lactobacillus*; (**F**) *Muribaculum*; (**G**) *Muribaculum-unclassified*. Values are mean ± SD (*n* = 5). *P < 0.05, **P < 0.01 versus NOR group; #*P* < 0.05, ##*P* < 0.01 versus HFD group.

Subsequently, gut microbiota compositions were analyzed among the three groups. As shown in Fig. 7F-I, compared with the NOR mice, the HFD-fed mice exhibited increased abundances of Firmicutes and Proteobacteria phyla, but a decreased abundance of Bacteroidota phylum. After treatment with GEN, the elevated abundances of Firmicutes and Proteobacteria phyla were notably reversed, and the reduced abundance of Bacteroidota was also increased compared with the HFD group. Furthermore, at the genus level, compared with NOR group, the HFD-fed mice exhibited increased abundance of genera such as *Ruminococcus*, *Allobaculum*, and *Helicobacter*. In contrast to the HFD-fed mice, the HGE group showed decrease in the aforementioned values, while the higher abundances of *Lactobacillus*, *Muribaculum*, and *Muribaculaceae_unclassified* were detected (*P* < 0.01, Fig. 8). These findings indicated that GEN might ameliorate the imbalance in gut microbiota in MASLD mice.

## Discussion

MASLD is a chronic metabolic disorder intricately associated with obesity and is commonly identified as a hepatic manifestation of metabolic syndrome. It may be related to genetic factors, metabolic abnormalities, and unhealthy lifestyles, including sedentary behavior and unhealthy diet. Hepatic lipogenesis and fatty liver induced by obesity are coordinated by extensive transcriptional pathways regulating carbohydrate and lipid metabolism^23^. Given the close association between MASLD and obesity, an MASLD mouse model was induced through a high-fat diet to evaluate the progression of this disease^24^. GEN is a naturally occurring phytoestrogen, which is known for its anti-inflammatory, anti-diabetic, and lipid-lowering effects^17,25^. However, it is still unclear whether GEN improves MASLD by regulating the gut microbiota. Therefore, comprehensive evaluation of its mechanisms is crucial for enhancing its potential as a therapeutic agent for MASLD. The present study confirmed our hypothesis that the intake of GEN improves in glucose-lipid metabolism and hepatic pathology in MASLD *via* regulating gut microbiota and hepatic macrophage polarization.

MASLD is commonly associated with obesity and metabolic disturbances, leading to increased systemic inflammation and impaired insulin signaling, thus promoting the development of various metabolic diseases, including MASH and T2DM^26^. SHRI is used for clinical quantification of hepatic steatosis and measurement of both severe and mild hepatic fat accumulation^22^. Elevation of AST and ALT reflects the extent of liver damage in MASLD^27^. The results indicated that MASLD mice had increased body weight and impaired liver function, as well as disrupted lipid metabolism and glucose metabolism, compared to the NOR mice. The liver histopathology of HFD mice showed typical features of MASLD, including hepatocellular ballooning, lipid droplet accumulation, and inflammatory infiltration. These results confirmed the successful establishment of the MASLD model in our study. Following GEN intervention, although the LGE group showed minimal significant improvements, the MGE and HGE groups showed significant improvements in these indices, demonstrating that GEN was able to ameliorate the disorders of glucose-lipid metabolism and liver function in MASLD.

Macrophages are the primary phagocytes in the liver, which are mainly divided into M1 and M2 phenotypes. M1 macrophages are often polarized by stimuli such as LPS, promoting inflammation by releasing pro-inflammatory mediators^28^. M2 macrophages exhibit anti-inflammatory characteristics by releasing anti-inflammatory mediators. In addition to resident macrophages, bone marrow-derived macrophages also play a crucial role not only in maintaining liver homeostasis but also in influencing the progression of liver pathologies^29^. There is a bidirectional interaction between macrophages and hepatocytes, which regulates lipid accumulation in the liver^30^. Hence, the accumulation of hepatic macrophages may trigger fat accumulation in the liver and promote the progression of MASLD to MASH. Inflammatory responses are significantly decreased by IL-10 from M2 macrophages in the periportal vein zones and the pericentral vein zones of liver^31^. Previous research has found that in HFD-induced MASLD animal models, excessive polarization of M1 macrophages and M1/M2 macrophages imbalance are closely related to liver damage^32^. Therefore, the M1/M2 imbalance exacerbates the progression of MASLD. The experimental data indicated that HFD feeding could upregulate the concentration of LPS in serum and induce an increase in M1 macrophage populations and a reduction in M2 macrophage populations in liver tissue, suggesting that the imbalance of macrophage polarization contributed significantly to the development of MASLD. After intervention with GEN, the LPS levels were significantly decreased, and liver pathological damages were improved in MASLD mice. Meanwhile, the experimental results also demonstrated that GEN could reduce M1 macrophages while increasing M2 macrophages, thereby correcting the M1/M2 imbalance and thus inhibiting further progression of MASLD.

LPS is a principal constituent of the cell wall of Gram-negative bacteria and plays a pivotal role in exacerbating inflammation. Following a sustained high-fat diet, pathogenic bacteria in the gut microbiota of mice proliferate, leading to excessive LPS production^33^. LPS can reduce the expression of tight junction proteins such as claudin-1 and occludin, thereby impairing the intestinal barrier function^34^. Subsequently, gut-derived LPS can translocate into the bloodstream through the damaged intestinal mucosa; then reach the liver via the portal vein, and induce hepatic inflammation, which in turn promotes the progression of MASLD. The experimental results demonstrated decreased expression of colonic tight junction proteins in HFD mice, resulting in increased serum LPS levels. GEN intervention could significantly increase the expression of tight junction proteins (claudin-1 and occludin) and reduce serum LPS levels, indicating that GEN could improve intestinal barrier integrity and alleviate hepatic inflammation.

A wide range of gastrointestinal disorders, including MASLD^35^, ulcerative colitis^36^, diabetes^37^, and obesity^21^, are intricately associated with the balance of intestinal microbiota. Disturbance of a healthy gut environment leads to gut microbiota imbalance, and the imbalance of microbiota contributes to the severity of MASLD^38^. The genus *Helicobacter*, belonging to the Proteobacteria phylum, has dual effects: it promotes inflammation and induces IR^39^. Decreased *Helicobacter* abundance is deemed advantageous for mitigating metabolic endotoxemia in obese mice^40^. Proteobacteria has been identified as a microbial biomarker for MASLD, with a significant correlation to liver prognosis^10,41^. Its elevated abundance is associated with the progression of HFD-induced liver fibrosis. The genus *Ruminococcus* is characterized by its intestinal pro-inflammatory effects. The abundance of *Allobaculum* is markedly augmented in HFD-induced obesity, and *Allobaculum* is speculated to be associated with the expression of lipid metabolism genes^42^. Firmicutes is positively correlated with steatosis in MASLD models^43^. Notably, these bacteria are part of the microbial network responsible for glycolytic metabolite synthesis. The proliferation of pathogenic bacteria in MASLD models induces the production of endogenous ethanol and ethanol-derived metabolites, which directly impair hepatic function, increase intestinal permeability, and elevate portal vein LPS levels^44^. The results showed that HFD induced an increase in Proteobacteria and Firmicutes, and a decline in Bacteroidota, further confirming the close association between gut microbiota dysbiosis and MASLD. After intervention with GEN, the abundances of Proteobacteria and Firmicutes decreased, while the abundance of Bacteroidota increased, indicating that GEN could regulate the gut microbiota in MASLD mice. At the genus level, *Lactobacillus* and *Muribaculum* were involved in reducing hepatic TG, TC, and LDL-C levels, which may be achieved by enhancing bile salt hydrolase activity and promoting the excretion of free bile acids in feces^44,45^. A study has discovered that gut microbiota, inflammatory cells, and inflammatory proteins affect MASLD and acute pancreatitis with LDL-C as a mediator^46^. Conversely, the quantity and type of lipids alter the composition and physiological functions of the gut microbiota. In the current study, the experimental findings showed that the development of MASLD was also associated with an increase in the abundance of *Helicobacter* (phylum Proteobacteria), *Ruminococcus* and *Allobaculum* (phylum Firmicutes). GEN intervention enhanced beneficial bacteria and reduced pathogenic bacteria, restoring gut microbiota homeostasis in MASLD. Hence, GEN has vast therapeutic potential, and future studies are required to explore its efficacy in preventing and treating MASLD through diet or supplement forms. Furthermore, the study of the synergistic effects of GEN with other natural compounds will also provide a new idea for more comprehensive treatment.

Although mouse models offer an important experimental tool for investigating MASLD, they have some limitations in simulating the complexity of human disease. For instance, mice and humans differ in gene expression, metabolic pathways, and immune responses, which may affect the extrapolation of the findings. Consequently, it is imperative to integrate both murine model research and population studies in order to ascertain the accuracy and reliability of the research findings.

## Conclusions

In summary, we explored the potential role of GEN in inhibiting the progression of MASLD. GEN supplementation enhanced the integrity of the intestinal mucosal barrier by reshaping gut microbiota composition, reduced serum LPS levels, and restored the polarization balance of hepatic M1/M2 macrophages, thereby improving hepatic metabolic function and highlighting that GEN holds potential as a natural health supplement for the management of MASLD.

## Supplementary Information

Below is the link to the electronic supplementary material.

Supplementary Material 1

## Data Availability

The data that support the findings of this study are available from the corresponding author upon reasonable request.
