# Supplementary material for: Genistein ameliorates non-alcoholic fatty liver disease in mice by modulating gut microbiota and hepatic macrophage polarization
Source: Sci Rep. 2025 Dec 18;15:44085. doi: 10.1038/s41598-025-27783-3 (PMC12714702; doi:10.1038/s41598-025-27783-3)
Supplement: Supplementary file 1 — Supplementary Material 1 [file 41598_2025_27783_MOESM1_ESM.doc]

**Supplementary file** **“ameliorates non-alcoholic fatty liver disease in mice by modulating gut microbiota and hepatic macrophage polarization”**

Shanshan Fang ^a, c^, Hui Han ^a^, Rui Wang ^a^, Siyu Li ^a^, Rui Yang ^b🖂^, Shomaila Mehmood ^d^, Qiang Jia ^a🖂^

^a^ School of Basic Medicine, Bengbu Medical University, Bengbu 233030, China

^b^ School of Biology and Food Engineering, Hefei Normal University, Hefei 230601, China

^c^ Huaibei People's Hospital, Huaibei 235000, China

^d^ Institute of Environmental Health Sciences, Wayne State University, Detroit 48201, USA

^🖂^Corresponding author:

Rui Yang. Email: xzxyr@sina.com.

Qiang Jia. Email: jiaq12@sina.com.

“

A

L 1 2 3 4 5 L


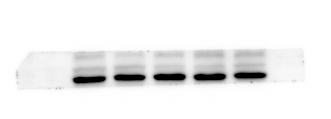


β-actin, 43 kDa

40kDa

40kDa


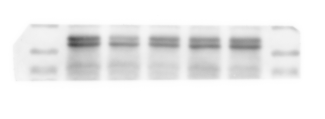


Arg1, 36 kDa

35kDa

35kDa

25kDa

25kDa


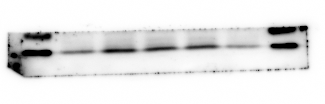


20kDa

20kDa

TNF-α, 17 kDa

15kDa

15kDa


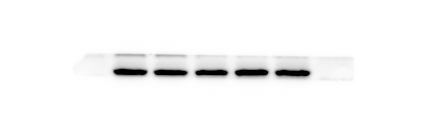


β-actin, 43 kDa

B

L 1 2 3 4 5 L


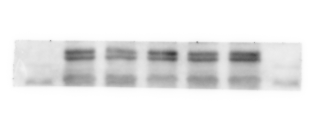


Arg1, 36 kDa


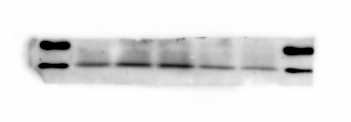


TNF-α, 17 kDa

L 1 2 3 4 5 L


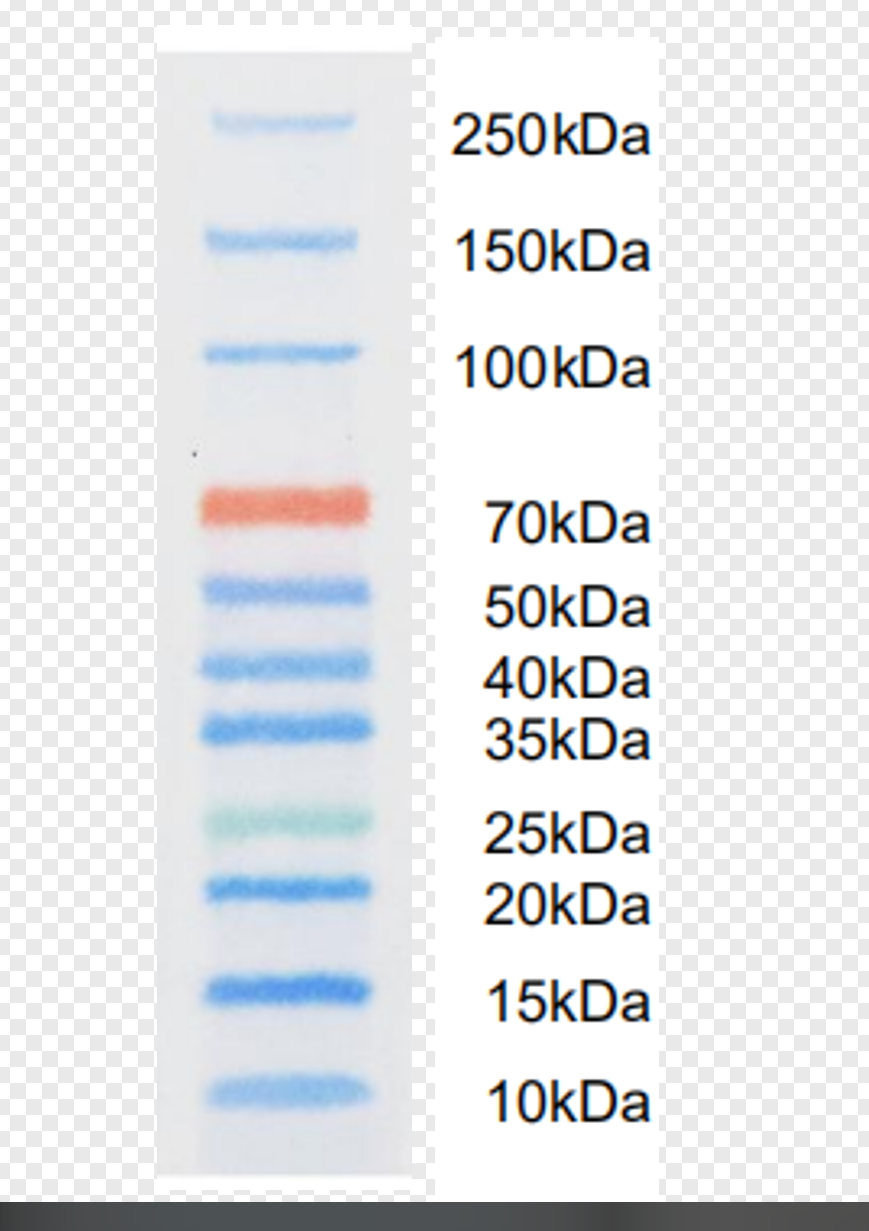


C


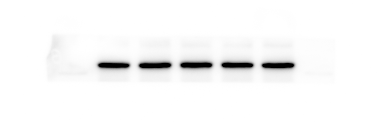


β-actin, 43 kDa


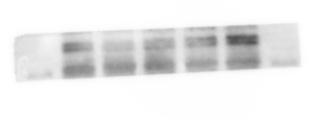


Arg1, 36 kDa


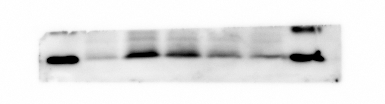


TNF-α, 17 kDa

**Supplementary Figure 1. Western blot images for β-actin and Arg1 and TNF-α in liver tissue.** The following lane sequence applies to all images, with L representing ladder: 1: NOR, 2: HFD, 3: LGE, 4: MGE, 5: HGE；A) Western blot results of Arg1 and TNF-α and β-actin. B) Replicate membrane for A，C) Replicate membrane for A.


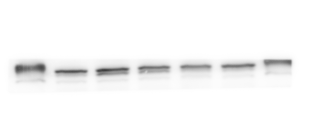

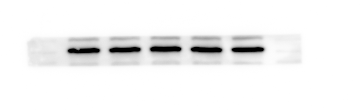

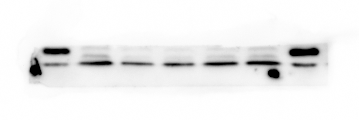

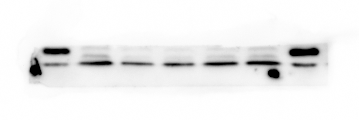


F


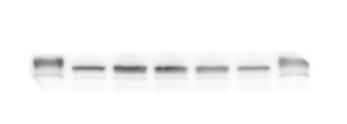

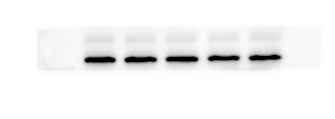

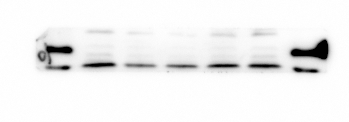


70kDa

iNOS, 65 kDa

β-actin, 43 kDa

IL-10, 19 kDa

40kDa

20kDa

15kDa

70kDa

40kDa

20kDa

15kDa

L 1 2 3 4 5 L

D


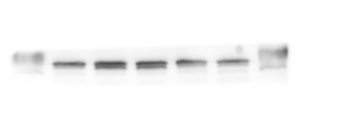

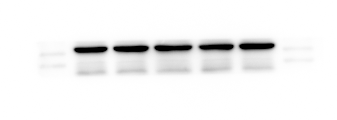

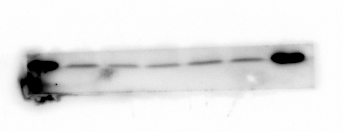


β-actin, 43 kDa

iNOS, 65 kDa

IL-10, 19 kDa

β-actin, 43 kDa

iNOS, 65 kDa

IL-10, 19 kDa

E

L 1 2 3 4 5 L

L 1 2 3 4 5 L

**Supplementary Figure 2. Western blot images for β-actin and iNOS and IL-10 in liver tissue.** The following lane sequence applies to all images, with L representing ladder: 1: NOR, 2: HFD, 3: LGE, 4: MGE, 5: HGE, D) Western blot results of IL-10 and iNOS and β-actin, E) Replicate membrane for D, F) Replicate membrane for D.
